# Novel protocol combining physical and nutrition therapies, Intensive Goal-directed REhabilitation with Electrical muscle stimulation and Nutrition (IGREEN) care bundle

**DOI:** 10.1186/s13054-021-03827-8

**Published:** 2021-12-04

**Authors:** Hidehiko Nakano, Hiromu Naraba, Hideki Hashimoto, Masaki Mochizuki, Yuji Takahashi, Tomohiro Sonoo, Yasuhiro Ogawa, Yujiro Matsuishi, Nobutake Shimojo, Yoshiaki Inoue, Kensuke Nakamura

**Affiliations:** 1 Department of Emergency and Critical Care Medicine, Hitachi General Hospital, 2-1-1 Jonancho, Hitachi, Ibaraki Japan; 2 Department of Emergency and Critical Care Medicine, Faculty of Medicine, University of Tsukuba, Tsukuba, Ibaraki Japan; 3 Neuroscience Nursing, St. Luke’s International University, Tokyo, Japan

**Keywords:** NMES, EGDM, Critical care nutrition, Nitrogen balance, ICU-AW, Titin

## Abstract

**Background:**

Although the combination of rehabilitation and nutrition may be important for the prevention of intensive care unit (ICU)-acquired weakness, a protocolized intervention of this combination has not yet been reported. We herein developed an original combined protocol and evaluated its efficacy.

**Methods:**

In this single-center historical control study, we enrolled adult patients admitted to the ICU. Patients in the control group received standard care, while those in the intervention group received the protocol-based intervention. The ICU mobility scale was used to set goals for early mobilization and a neuromuscular electrical stimulation was employed when patients were unable to stand. The nutritional status was assessed for nutritional therapy, and target calorie delivery was set at 20 or 30 kcal/kg/day and target protein delivery at 1.8 g/kg/day in the intervention group. The primary endpoint was a decrease in femoral muscle volume in 10 days assessed by computed tomography.

**Results:**

Forty-five patients in the control group and 56 in the intervention group were included in the analysis. Femoral muscle volume loss was significantly lower in the intervention group (11.6 vs 14.5%, *p* = 0.03). The absolute risk difference was 2.9% (95% CI 0.1–5.6%). Early mobilization to a sitting position by day 10 was achieved earlier (*p* = 0.03), and mean calorie delivery (20.1 vs. 16.8 kcal/kg/day, *p* = 0.01) and mean protein delivery (1.4 vs. 0.8 g/kg/day, *p* < 0.01) were higher in the intervention group.

**Conclusion:**

The protocolized intervention, combining early mobilization and high-protein nutrition, contributed to the achievement of treatment goals and prevention of femoral muscle volume loss.

***Trial registration number*:**

The present study is registered at the University Hospital Medical Information Network-clinical trials registry (UMIN000040290, Registration date: May 7, 2020).

**Supplementary Information:**

The online version contains supplementary material available at 10.1186/s13054-021-03827-8.

## Introduction

Intensive care unit (ICU)-acquired weakness (AW) has a significant impact on the course of patients during the ICU stay, hospitalization, and after discharge, and efforts to minimize it have been examined in recent years [1]. ICU-AW is caused by various factors [2], including muscle volume loss mainly in the ubiquitin–proteasome system [3], and muscle weakness associated with decreased muscle activity [4] and sarcomere structural changes [5], both of which are major issues [6]. Muscle volume loss and muscle weakness do not necessarily coincide [7], and, thus, need to be evaluated separately. Although evidence to support the prevention of ICU-AW by early mobilization is limited, previous studies indicated that early mobilization improved physical function by hospital discharge [8, 9] and facilitated recovery from ICU-AW [10]. In addition, the attenuation of muscle volume loss and muscle weakness by automatic exercise, such as NMES, has been reported [11–14] and advocated as an approach to minimize ICU-AW under conditions in which usual early mobilization is difficult [15]. Not only rehabilitation but also nutritional therapy is important for minimizing ICU-AW. The achievement of a target calorie level during an ICU stay contributes to patient outcomes, with 70–80% underfeeding management in the ICU being optimal [16]. Some argue that nutritional therapy needs to be assessed with regard to target muscle volume and strength [17]. Furthermore, protein administration is essential for maintaining muscle volume [18] and is regarded as an important target [19]; it is associated with patient outcomes [16, 20, 21], but is challenging to achieve, particularly with underfeeding management [22, 23].

Protocolized interventions of rehabilitation and nutrition therapy may be important as a specific approach to achieve these goals. Previous studies demonstrated that early mobilization was achieved by protocolized interventions [24, 25]. Furthermore, the effectiveness of early goal-directed mobilization (EGDM) was recently reported in postoperative patients [26] and patients on ventilators [27]. In terms of nutrition, the PEPuP protocol [28] and EFFORT trial [29] achieved improvements through nutritional goals by protocolization. Protocolized interventions may provide a more detailed mutual understanding among professions and facilitate collaborations, making it easier to achieve treatment goals.

The combination of appropriate exercise and nutrition maximizes the effects of training in healthy individuals [30], and the combination of exercise and nutrition has also been applied to critically ill patients or during their recovery [31–33]. Therefore, their coordination may also be important for the prevention of ICU-AW [19, 34–36]. Although it is desirable to use a protocol that combines these interventions, this has not yet been achieved.

Another limitation is that it is difficult to assess muscle injury in real time. N-titin is a biomarker of muscle injury that may be measured in urine [37, 38], was previously shown to be associated with muscle weakness in critically ill patients, and is expected to become a new biomarker [39, 40].

We herein developed the Intensive goal-directed rehabilitation with electrical muscle stimulation and nutrition (IGREEN) protocol, a unique protocol that combines early mobilization and high protein nutrition therapy, and conducted a historical control study. To verify the efficacy of the protocol to ameliorate muscle injury in ICU-AW, we assessed muscle volume as the primary endpoint and physical function as the secondary endpoint. We also measured N-titin to assess its significance as an indicator of treatment efficacy in these patients.

## Methods

### Study design and patients

After Ethics Committee approval (2020-38), this single-center, historical control study was conducted at Hitachi General Hospital. It is registered at the University Hospital Medical Information Network-clinical trials registry (UMIN000040290). Patients admitted to the ICU and managed by our department between September 2020 and December 2020 were included in the intervention group. Exclusion criteria were younger than 20 years old, possible pregnancy, anuria, a lower extremity perturbation, such as infection, injury, or amputation, the use of extracorporeal membrane oxygenation, expected to be discharged from the ICU within 2 days, admission to the ICU for a second time during the same hospital stay, and the designation of “do not attempt resuscitation”. Informed consent was provided by each eligible participant or a proxy. Patients admitted to the ICU between October 2019 and February 2020, treated with standard care, and who underwent the same evaluation as the intervention group were included in the control group. Patients in the standard care group were the subjects of a previous observational study [39] and were evaluated in the same manner as that described in the present study after consent was obtained. In the present study, we obtained approval from the Ethics Committee to waive consent for patients in the standard care group.

### Protocol

**Fig. 1:**
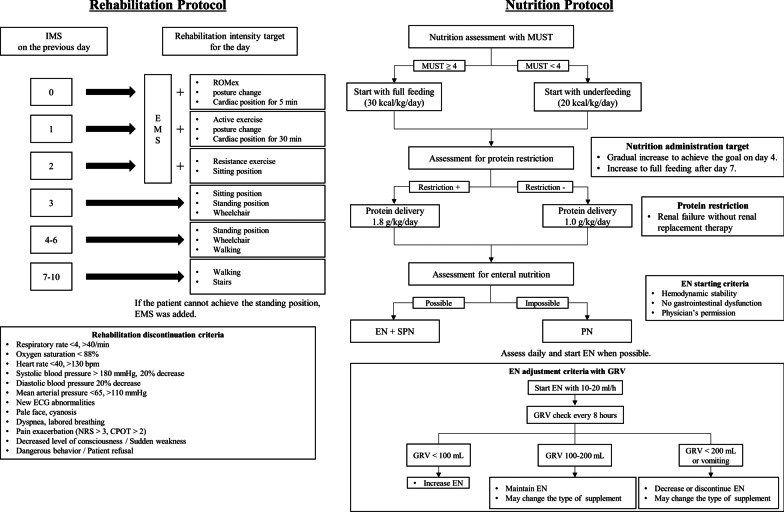
IGREEN protocol. In the evaluation of MUST, in addition to the 2 points for acute illness, 1 point was given for BMI ≤ 20 and 2 points for BMI ≤ 18.5, and 1 point was given for weight loss ≥ 5% and 2 points for weight loss ≥ 10% in the 6 months prior to admission, as confirmed by interviews with the patients’ families. *IMS* intensive care unit mobility scale, *EMS* electrical muscle stimulation, *ROMex* range of motion exercise, *ECG* electrocardiogram, *NRS* numerical rating scale, *CPOT* critical care pain observation tool, *MUST* malnutrition universal screening tool, *EN* enteral nutrition, *SPN* supplemental parenteral nutrition, *GRV* gastric residual volume

An overview of the IGREEN protocol is shown in Fig. 1.

#### Rehabilitation

The first morning after entering the ICU was defined as day 1, and rehabilitation began after entering the ICU in both groups. Physical therapists only intervened on weekdays, and nurses performed rehabilitation in the bed on weekends. Rehabilitation was conducted when there was no conflict with the criteria for the discontinuation of rehabilitation, including unstable vital signs, new electrocardiographic changes, severe pain, impaired consciousness, and patient refusal (Fig. 1). No specific goals were set for the standard care group and NMES was not performed. While in the ICU, rehabilitation was performed in the bed for 20 min a day, including cardiac and sitting positions. In the intervention group, goals were set using the ICU mobility scale (IMS) [41, 42] to achieve EGDM. Each morning, a multidisciplinary conference was held to share goals and safely execute mobilization. If there were no restrictions, the intervention was targeted to achieve a higher level of IMS than the previous day. Although the IGREEN protocol used IMS as the mobilization assessment, the intervention method as EGDM was similar to that of the SOMS protocol [26], not the IMS-based protocol of previous studies [27]. In addition, in the IGREEN protocol, NMES was performed on days when IMS 4, which corresponds to the standing position, was not achieved. We used the belt-type NMES, GTES® (Homer Ion Co., Ltd., Tokyo, Japan) and the same protocol as that in our previous study [33]. Belt-type electrodes were attached to the trunk and lower extremities. NMES was performed on the entire lower body once a day for 20 min at a frequency of 20 Hz with a pulse width of 250 ms. The on: off time was set to 5 s of stimulation with a 2-s pause. Electrical intensity was adjusted by a physical therapist according to muscle contraction and patient discomfort, expressions, or vital sign changes.

#### Nutrition

In the absence of contraindications, enteral nutrition (EN) was started in both groups via a nasogastric tube within 48 h of admission at 10–20 mL/h and gradually increased. In the standard care group, nutritional therapy was given at the discretion of the attending physician according to the patient’s condition and severity. In the intervention group, a nutritional assessment was performed using the malnutrition universal screening tool (MUST) [43] at admission. In the evaluation of MUST, in addition to the 2 points for acute illness, 1 point was given for BMI ≤ 20 and 2 points for BMI ≤ 18.5, and 1 point was given for weight loss ≥ 5% and 2 points for weight loss ≥ 10% in the 6 months prior to admission, as confirmed by interviews with the patients’ families. In the absence of malnutrition (MUST < 4), EN was gradually increased to deliver 20 kcal/kg/day. In the presence of malnutrition (MUST ≥ 4), it is considered to set a target calorie delivery for day 4 at 30 kcal/kg/day. After day 7, target calorie delivery for all patients was 30 kcal/kg/day. Target protein delivery was set at 1.8 g/kg/day, or 1.0 g/kg/day when protein restriction was necessary. Any shortage was compensated for by supplemental parenteral nutrition and intravenous amino acids were also used. EN was adjusted using the gastric residual volume as a guide (Fig. 1). The enteral formula was changed or discontinued when patients developed diarrhea of Bristol stool scale > 5 [44]. Actual body weight was used for nutritional calculations; however, in the case of body mass index ≥ 30, body height (m)* body height (m)*25 was used. Oral nutrition supplements were provided in addition to the hospital diet when patients recovered and were able to take food orally. This intervention was continued until day 10.

### Outcomes

#### Primary endpoint

The primary endpoint was set as femoral muscle volume (FMV) loss (%) during the first 10 days after ICU admission. In both groups, plain femoral CT was performed on days 1 and 10. On day 10, CT was performed even if the patient was discharged from the ICU to the general ward. The CT scanning protocol was the same as that described in our previous studies. CT scanning parameters were as follows: 64-/128-slice CT with 120 kV tube voltage, 150–600 mA tube current (auto exposure control), 0.35 s scan time, 0.625 × 64 collimation, 1.078 table pitch, and 2.5 mm slice thickness, using our dedicated CT scanner located in the Emergency center (Scenaria; Hitachi Ltd., Tokyo, Japan). Scanning was performed from the femoral head to the patella, avoiding pelvic organs as much as possible. Images were analyzed using a system volume analyzer (Vincent®; Fujifilm Corp., Tokyo, Japan). All femoral muscles were extracted with CT values of 0–100. The femoral muscle volume (mL) was calculated using the sagittal direction integration of the cross-sectional area of the femoral muscle. The estimated exposure radiation dose was calculated as a maximum of 10–18 mGy using Waza-ari, a web-based CT dose calculation program. These analyses were conducted by a radiology technician blinded to the contents of the study. Patients judged by the radiology technician to have inappropriate measurements, such as severe edema or difficulty with taking measurements according to the protocol due to flexion of the lower limbs, were excluded from analyses.

#### Secondary endpoints

The secondary endpoints related to rehabilitation were the dates of achievement of IMS 3 and IMS 4, Medical Research Council (MRC) scores, grip strength and functional status scores for the ICU (FSS-ICU) at ICU discharge, and the Barthel Index at hospital discharge. Physical function was evaluated by trained physical therapists, and was recorded as zero for patients who died as previously reported [8].

Calorie and protein deliveries, the nitrogen balance, and the proportion of EN failure were evaluated as nutrition outcomes. Daily total calorie and protein deliveries were calculated by a hospital nutritionist. Nutrition intake in patients taking food orally was assessed using hospital ready reckoners and the recorded food intake. Nitrogen balance was calculated using a 24-h urine collection as follows: Nitrogen balance = Protein delivery (g/day)/6.25 − Total urine nitrogen (g/day) × 1.25 [45]. EN failure was defined as the discontinuation of EN due to diarrhea, vomiting, or a high gastric residual volume.

Secondary endpoints related to treatment included the proportion of survival discharge, the lengths of ICU and hospital stays, and the use of adjunctive therapy, such as mechanical ventilation, renal replacement therapy, steroids, and continuous neuromuscular blocking agents. Steroid doses were recorded as the total amount of a hydrocortisone equivalent within 10 days.

We also evaluated the mean values of and changes in N-titin/Cre from days 1 to 7 and laboratory data on day 10. N-titin was measured using an enzyme-linked immunosorbent assay kit (#27900 Human Titin N-Fragment Assay Kit; Immuno-Biological Laboratories, Fujioka, Japan) as described in our previous study [39], and we used the value of the spot urine N-titin level divided by the spot urine creatinine concentration and by 10 (N-titin/Cre) (pmol/mgCre). We recorded blood urine nitrogen and creatinine as indicators of renal function, albumin and the total lymphocyte count as indicators of the nutritional status [46], and C-reactive protein as an indicator of inflammation.

Age, sex, body height, body weight, sequential organ failure assessment scores, acute physiology and chronic health evaluation II scores, the Charlson comorbidity index, MUST, the ratio of patients with difficulty walking before admission, the main diagnosis, and laboratory data on day 1 were recorded as basic characteristics.

To evaluate the factors associated with FMV loss, we conducted an exploratory multivariate linear regression analysis of FMV loss. The relationship between N-titin/Cre and various physical functions were examined, and diagnostic accuracy for MRC < 48 was also confirmed.

### Statistical analysis

We assessed the normality of the distribution using the Shapiro–Wilk test. Parametric continuous variables were expressed as means and standard deviations, and non-parametric continuous variables as medians and interquartile ranges. Comparisons between the two groups were performed using the Student’s *t*-test for parametric continuous variables, the Mann–Whitney *U* test for non-parametric variables, and the chi-squared test for categorical variables. To assess early mobilization, Kaplan–Meier curves were drawn for the proportion of patients who achieved IMS 3 and IMS 4 by day 10, and differences were assessed with the Log-rank test. In the multivariate linear regression analysis, we selected age, sex, and items that correlated in the univariable linear regression analysis as explanatory variables. A two-way repeated measures analysis of variance was used to compare the two groups for daily N-titin/Cre. To confirm the diagnostic accuracy of N-titin/Cre for MRC < 48, a receiver operator characteristic (ROC) curve was drawn, the area under ROC (AUROC) was calculated, and the value with the highest accuracy was set as the cut-off value. A *p*-value < 0.05 was considered to indicate a significant difference. Statistical analyses were performed using R (version 4.0.0., R Foundation for Statistical Computing, Vienna, Austria).

## Results

**Fig. 2:**
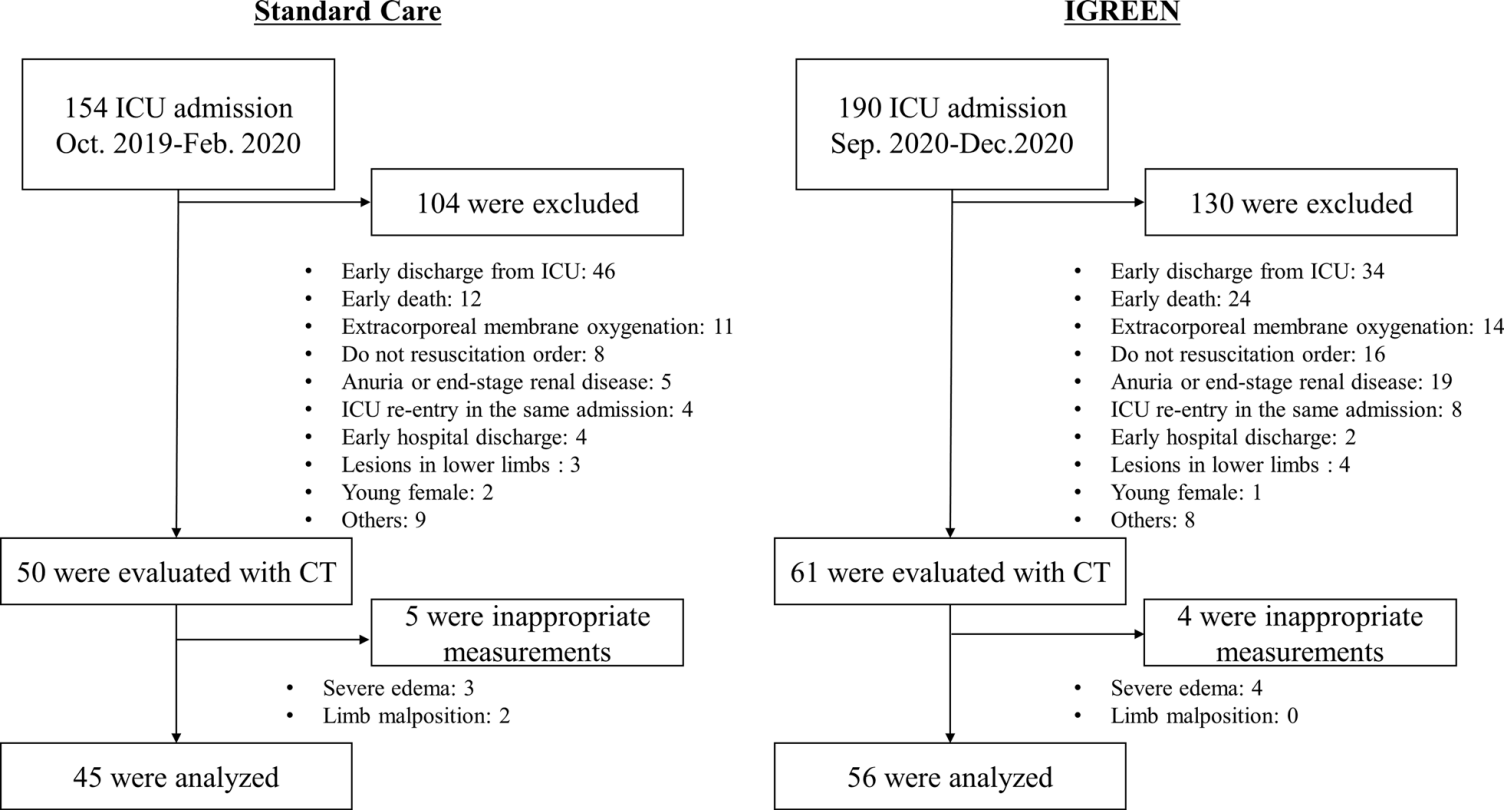
Patient selection. *IGREEN* Intensive goal-directed rehabilitation with electrical muscle stimulation and nutrition protocol, *ICU* intensive care unit, *CT* computed tomography

Patient selection is shown in Fig. 2. The final analysis included 45 patients in the standard care group and 56 in the intervention group. Basic characteristics are shown in Table 1. No significant differences were observed in age, sex, height, weight, the nutritional status, or the ratio of patients with difficulty walking before admission. Furthermore, no significant differences were noted in the severity of illness or comorbidities. The results of blood collection on day 1 did not significantly differ between the two groups.

**Table 1:** Basic characteristics

	IGREEN	Standard care	*p* value
	*n *= 56	*n* = 45	
Age, yr	70.9 (14.5)	70.9 (14.2)	1
Sex, M, *n* (%)	39 (69.6)	34 (75.6)	0.66
Body height, cm	162.4 (9.5)	162.9 (11.7)	0.82
Body weight, kg	59.0 (12.6)	57.6 (13.5)	0.60
SOFA	7.9 (2.9)	7.8 (3.1)	0.93
APACHE II	17.7 (6.5)	16.0 (5.7)	0.17
CCI, median (IQR)	1.0 (1.0, 2.0)	1.0 (0.0, 2.0)	0.33
Difficulty walking before admission, *n* (%)	6 (10.7)	3 (6.7)	0.72
MUST	2.6 (1.0)	2.6 (0.9)	0.85
preICU hospital stay, d, median (IQR)	0.0 (0.0, 0.0)	0.0 (0.0, 0.0)	0.45
Diagnosis, *n* (%)			0.48
Cardiopulmonary arrest	4 (7.1)	4 (8.9)	
Cardiovascular	4 (7.1)	6 (13.3)	
Endocrine and metabolic disorders	5 (8.9)	1 (2.2)	
GI bleeding	1 (1.8)	0 (0.0)	
Post-surgery	3 (5.4)	4 (8.9)	
Respiratory failure	6 (10.7)	2 (4.4)	
Sepsis	24 (42.9)	18 (40.0)	
Stroke	2 (3.6)	5 (11.1)	
Trauma	7 (12.5)	5 (11.1)	
Laboratory data on day 1			
BUN, mg/dL, median (IQR)	23.4 (18.0, 38.4)	26.8 (17.7, 45.5)	0.51
Creatinine, mg/dL, median (IQR)	1.0 (0.8, 1.6)	1.2 (0.8, 1.7)	0.45
Albumin, mg/dL	3.0 (0.6)	2.9 (0.6)	0.63
TLC, /mm^3^, median (IQR)	819 (570, 1092)	678 (392, 1085)	0.21
CRP, mg/dL, median (IQR)	4.3 (1.4, 8.4)	6.4 (2.2, 14.8)	0.15

*SOFA* sequential organ failure assessment score, *APACHE II* acute physiological and chronic health evaluation II score, *CCI* Charlson comorbidity index, *MUST* malnutrition universal screening tool, *BUN* blood urea nitrogen, *TLC* total lymphocyte count

Data are shown as means (SD) unless otherwise noted

**Fig. 3:**
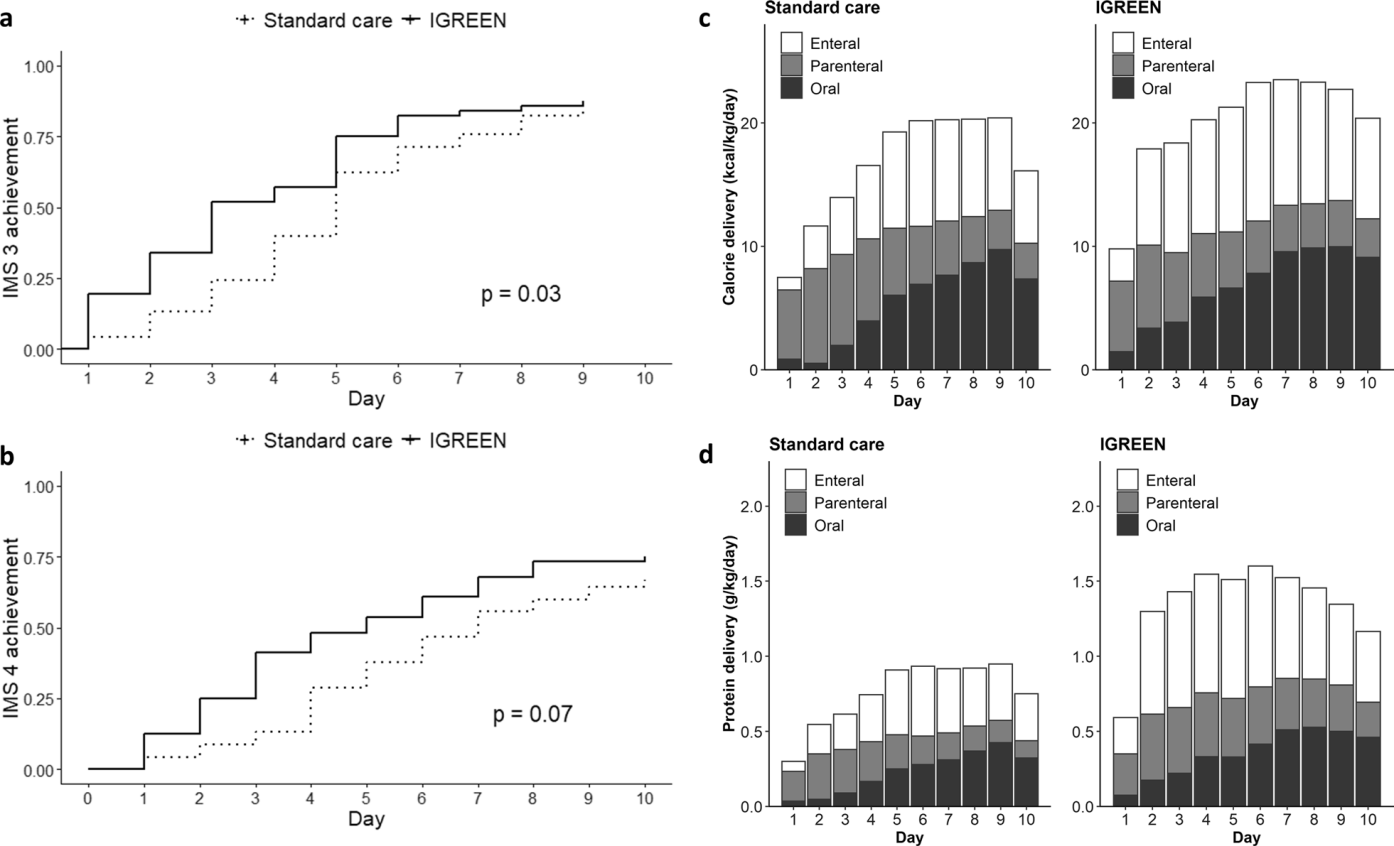
Comparison of therapeutic outcomes in rehabilitation and nutrition. **a** Kaplan–Mayer curves for IMS 3, **b** Kaplan–Mayer Curves for IMS 4, c daily trajectory of mean calorie delivery, and **d** daily trajectory of mean protein delivery. The Kaplan–Meier curve showed that the intervention group achieved IMS 3 earlier (*p* = 0.03), whereas no significant difference was observed in the achievement of IMS 4 (*p* = 0.07). Mean calorie delivery (20.1 vs. 16.8 kcal/kg/day, *p* = 0.01) and mean protein delivery (1.4 vs. 0.8 g/kg/day, *p* < 0.01) from days 1 to 10 were higher in the intervention group. Protein delivery was higher in the intervention group for both enteral nutrition (0.6 vs 0.3 kcal/kg/day, *p* < 0.01) and parenteral nutrition (0.4 vs 0.2 g/kg/day, *p* = 0.05). No significant difference was noted in mean calorie delivery (6.8 vs. 5.4 kcal/kg/day, *p* = 0.12) or mean protein delivery (0.4 vs. 0.2 g/kg/day, *p* = 0.44) for oral intake. *IMS* intensive care unit mobility scale, *IGREEN* intensive goal-directed rehabilitation with electrical muscle stimulation and nutrition protocol

The outcomes are shown in Table 2. The primary endpoint of FMV loss was significantly lower in the intervention group (11.6 vs 14.5%, *p* = 0.03). The absolute risk difference was 2.9% (95% CI 0.1–5.6%). Regarding rehabilitation of the intervention group, NMES was performed on all patients, except for two after the implantation of pacemakers, and mobilization was also conducted according to the protocol. NMES was performed 2.0 (1.8–4.0) times during the first 10 days, including after ICU discharge. The achievement rate of the protocol for NMES was 88.5% during the ICU stay and 76.2% during the first 10 days, including after ICU discharge. The number of days to achieve IMS 1 did not significantly differ between the two groups. The Kaplan-Meyer curve (Fig. 3) for the achievement of IMS3 by day 10 showed that the intervention group achieved earlier mobilization. However, no significant differences were observed in physical function, such as MRC scores and FSS-ICU at ICU discharge, or the Barthel Index at hospital discharge. In terms of nutritional therapy, two patients (3.6%) in the intervention group were targeted with a calorie delivery of 30 kcal/kg/day, and protein delivery was not restricted in any patient. Mean calorie delivery (20.1 vs. 16.8 kcal/kg/day, *p* = 0.01) and mean protein delivery (1.4 vs. 0.8 g/kg/day, *p* < 0.01) from days 1 to 10 were higher in the intervention group. Daily nutrition delivery by the route of administration is shown in Fig. 3. Protein delivery was higher in the intervention group for both EN (0.6 vs 0.3 kcal/kg/day, *p* < 0.01) and supplemental parenteral nutrition (0.4 vs 0.2 g/kg/day, *p* = 0.05). Regarding oral intake, no significant differences were observed in mean calorie delivery (6.8 vs. 5.4 kcal/kg/day, *p* = 0.12), mean protein delivery (0.4 vs. 0.2 g/kg/day, *p* = 0.44), or the date of first oral intake (4.0 vs. 4.0 days, *p* = 0.96). The cumulative nitrogen balance did not significantly differ, although nitrogen debt was slightly less in the intervention group. EN failure did not significantly differ between the two groups. No significant differences were observed in the proportion of survival discharge or lengths of hospital and ICU stays. Furthermore, adjunctive therapies did not significantly differ.

**Table 2:** Outcomes

	IGREEN	Standard care	*p* value
	*n* = 56	*n* = 45	
Primary endpoint			
Femoral muscle volume			
Day1, mL, median (IQR)	3730 (2268, 4573)	3230 (2410, 4640)	0.49
Day10, mL, median (IQR)	3145 (2298, 4220)	2810 (1960, 4230)	0.38
Loss from day 1 to 10, %	11.6 (5.9)	14.5 (7.6)	0.03*
Secondary endpoint			
Rehabilitation			
IMS 1 achieved, d, median (IQR)	1.0 (1.0, 2.0)	1.0 (1.0, 2.0)	0.54
IMS 3 achieved, d, median (IQR)	3.0 (2.0, 5.0)	5.0 (3.0, 5.8)	0.01*
IMS 4 achieved, d, median (IQR)	3.0 (2.0, 6.0)	5.0 (4.0, 7.0)	0.02*
MRC score, median (IQR)	49.5 (23.0, 58.0)	55.0 (37.0, 60.0)	0.26
Grip strength, kg, median (IQR)	14.1 (0.0, 20.1)	15.0 (0.0, 24.0)	0.8
FSS-ICU, median (IQR)	9.0 (3.0, 19.0)	9.5 (1.0, 19.5)	0.99
Barthel Index, median (IQR)	52.5 (5.0, 93.8)	70.0 (0.0, 100.0)	0.64
Nutrition			
Mean calorie delivery, kcal/kg/day	20.1 (5.7)	16.6 (5.6)	< 0.01*
Mean protein delivery, g/kg/day	1.4 (0.4)	0.8 (0.3)	< 0.01*
Cumulative nitrogen balance	− 27.8 (44.8)	− 42.1 (57.1)	0.16
EN failure, *n* (%)	4 (7.1)	4 (8.9)	1
Treatment			
Survival discharge, *n* (%)	50 (89.3)	39 (86.7)	0.92
Length of hospital stay, d, median (IQR)	29.0 (14.0, 63.0)	23.0 (16.0, 35.0)	0.27
Length of ICU stay, d, median (IQR)	6.0 (4.0, 9.0)	5.0 (4.0, 8.0)	0.59
MV use, *n* (%)	44 (78.6)	34 (75.6)	0.9
Duration of MV, d, median (IQR)	3.0 (1.0, 5.0)	2.0 (1.0, 5.0)	0.33
RRT use, *n* (%)	13 (23.2)	12 (26.7)	0.87
Steroid dose, mg, median (IQR)^†^	0.0 (0.0, 600.0)	0.0 (0.0, 600.0)	0.67
NMBA use, *n* (%)	0.0 (0.0, 0.0)	0.0 (0.0, 0.0)	0.82
N-titin/Cre, pmol/mgCre			
Mean, median (IQR)	96.3 (41.1, 181.5)	46.2 (21.9, 87.5)	< 0.01*
Change, median (IQR)	− 27.2 (− 166.7, − 1.0)	4.5 (− 26.1, 28.7)	< 0.01*
Laboratory data on day 10			
BUN, mg/dL, median (IQR)	36.6 (26.3, 50.7)	27.6 (17.6, 40.8)	0.02*
Creatinine, mg/dL, median (IQR)	0.9 (0.7, 1.2)	0.9 (0.6, 1.6)	0.94
Albumin, mg/dL, median (IQR)	2.8 (0.7)	2.6 (0.5)	0.25
TLC, /mm^3^, median (IQR)	1188 (808, 1634)	1125 (803, 1531)	0.5
CRP, mg/dL, median (IQR)	2.5 (0.7, 6.0)	2.7 (1.0, 5.1)	0.62

Data are shown as means (SD) unless otherwise noted

*ICU* intensive care unit, *MRC* Medical Research Council, *FSS-ICU* functional status score for the intensive care unit, *EN* enteral nutrition, *MV* mechanical ventilation, *RRT* renal replacement therapy, *NMBA* neuromuscular blocking agent, *N-titin/Cre* titin N-fragment in urine divided by urine creatinine, *BUN* blood urea nitrogen, *Cre* creatinine, *TLC* total lymphocyte count, *CRP* C-reactive protein

*p < 0.05, ^†^Total dose of a hydrocortisone equivalent within 10 days

The mean value of N-titin/Cre, a marker of muscle injury, was higher in the intervention group, but decreased significantly more from days 1 to 7 (Table 2). In A two-way repeated measures analysis of variance test, N-titin/Cre was higher in the intervention group on days 1 and 3, but not on days 5 and 7 (Additional file 1: Figure S1).

Regarding renal function, blood urea nitrogen on day 10 was higher in the intervention group, whereas serum creatinine did not significantly differ. No significant differences were observed in other laboratory data.

The results of the univariate linear regression revealed that FMV loss correlated with mean IMS, the cumulative nitrogen balance, the duration of mechanical ventilation, and mean N-titin/Cre (Additional file 1: Table S2). The multivariate logistic regression analysis revealed an independent relationship with mean IMS ((adjusted OR 0.35, 95% CI [0.18–0.67], *p* < 0.01) and the cumulative nitrogen balance (adjusted OR 0.96, 95% CI [0.94–0.99], *p* < 0.01), but not with N-titin/Cre (adjusted OR 1.00, 95% CI [1.00–1.01], *p* = 0.57) (Additional file 1: Table S2).

N-titin/Cre and physical function in all patients correlated with MRC scores (*ρ* = − 0.42, *p* < 0.01), FSS-ICU (*ρ* = − 0.47, *p* < 0.01), grip strength (*r* = − 0.25, *p* = 0.02), and the Barthel Index (*ρ* = − 0.25, *p* = 0.01) (Additional file 1: Figure S3). The AUROC of N-titin/Cre for MRC < 48 was 0.772 (0.681–0.864), with a sensitivity of 45% and specificity of 93% at a cut-off value of 159 pmol/mgCre (Additional file 1: Figure S4).

## Discussion

As recommended in recent years [19, 34–36] interventions with our combined protocol of rehabilitation and nutrition (IGREEN protocol) reduced FMV loss during the first 10 days of ICU admission. Early mobilization and calorie and protein delivery goals were achieved by the intervention. In the exploratory multivariate analysis, early mobilization and nitrogen balance were independently associated with FMV loss. N-titin correlated with physical function, but not with FMV loss. N-titin was higher in the intervention group than in the control group at ICU admission, but decreased significantly more in the first week, and the severity of illness did not significantly differ.

### Efficacy of protocolized intervention

In the present study, the absolute difference in FMV loss was approximately 3%. However, FMV loss in the standard care group (14.5%) was smaller than that in the non-intervention group in our previous study [11, 33, 47] and another study [3], suggesting that even the standard care group received high-quality care. Nevertheless, we were able to significantly attenuate FMV loss in the present study, which suggests the effectiveness of the intervention with the IGREEN protocol.

### Early mobilization

The IGREEN protocol achieved earlier mobilization at the IMS 3 level than standard care, which directly suggests the importance of protocolization. Furthermore, the early use of NMES may have further facilitated the prevention of FMV loss within 10 days, which is consistent with our previous findings [11]. Although it was not evaluated in the present study, Grunow et al. reported that the difference in contractile responses to NMES reflects muscle strength [48], and NMES may be useful as a tool for evaluating muscle injury as well as treatment in the future. However, the present results did not directly show a significant improvement in physical function due to the intervention, which may be attributed to the timing of the evaluation differing for each patient because it was set at the time of ICU or hospital discharge. A long-term follow-up and analysis of physical function, including recovery after discharge or transfer from the hospital, may lead to a more accurate evaluation. Additionally, the higher values of N-titin/Cre by day 3 in the intervention group may have influenced the present results.

### Nutrition therapy

The intervention with the IGREEN protocol allowed us to increase protein delivery even under the management of underfeeding. In addition, renal function did not worsen with high protein delivery, which is consistent with the recommendations by the ESPEN guidelines [49]. Although the overall average protein target of 1.8 g/kg/day was not achieved, average protein delivery was increased to more than 1.5 g/kg/day after day 4. The gradual increase in protein delivery after day 3 was considered to improve the prognosis of patients [19, 50], and the ESPEN guidelines recommend a target of at least 1.3 g/kg/day after the gradual increase [51], which was achieved in the intervention group. Although optimal protein delivery remains controversial, a higher target may need to be set because it was still difficult to achieve the target even with the protocol intervention. On the other hand, the intervention in the present study did not improve the nitrogen balance. Since the nitrogen balance is independently associated with FMV loss, it may be useful as a guide for protein delivery. Singer et al. recently reported the effectiveness of nutritional therapy using nitrogen excretion as a guide [52], and further studies on how the nitrogen balance may be used in clinical practice are expected. Protein delivery on days 2 and 3 was higher than the target in the present study, even though we aimed for a progressive increase until day 4. Previous studies reported that high protein delivery during the early period of the acute phase may be detrimental to muscle [53, 54], and that high protein doses before day 3 may worsen the prognosis of patients [50]. In the present study, a protein overdose in the early period may have had a negative impact with no significant improvements in physical function. In the early period, overdoses need to be avoided, and team building is important for the close monitoring of daily nutritional provision.

### N-titin/Cre

The results of the present study confirmed that N-titin correlated with physical function and may be used as a biomarker for ICU-AW. The intervention group had higher levels of N-titin and may have been at a higher risk of ICU-AW. Previous studies reported that high-protein administration during the early period of the acute phase may be detrimental to muscles [50, 51]; therefore, high-protein administration until day 3 in the intervention group may have worsened muscle injury. However, since N-titin/Cre on day 1 was assessed in urine before the start of nutritional therapy, high N-titin/Cre on day 1 may reflected muscle injury that could not be assessed by disease severity alone, rather than the effects of high-protein nutrition. Although the decrease in N-titin during the first week was significantly greater in the intervention group, it currently remains unclear whether this was an effect of the intervention, and, thus, further studies are needed.

### Limitations

There are some limitations that need to be addressed. First, this was a single-center study; therefore, selection bias cannot be excluded. A multicenter randomized controlled study is needed for a more accurate evaluation. Since data for a suitable cohort for the standard care group already existed, we herein conducted a historical control study. Although no significant differences were observed in demographic characteristics, the number of patients admitted to the ICU differed due to the difference in time periods between the standard care group and intervention group, which resulted in different numbers of patients being analyzed. However, the proportion of patients included in the analysis did not significantly differ from that admitted to the ICU. In addition, in terms of nutrition, differences were observed not only in protein delivery, which was the main target, but also in calorie delivery, which caused a limitation in that it was difficult to evaluate the effects of high-protein nutrition alone. Nevertheless, the calorie goal was also achieved by the protocol intervention, suggesting its importance. Moreover, nutrition therapy after the initiation of oral intake was inadequate. In terms of rehabilitation, we cannot show further details of rehabilitation dosing. The lack of assessment of muscle contraction while NMES as an objective assessment of rehabilitation intensity or muscle strength is also a limitation. Another limitation is that the effects of treatment after day 10 were not assessed and may have affected physical function at hospital discharge. It is also important to combine rehabilitation and nutrition therapy during the recovery period and strengthen collaborations between these professions in the future. In addition, the long-term prognosis of patients was not evaluated in the present study. Although no significant differences were observed in physical function during hospitalization, the prevention of FMV loss may have contributed to a better long-term prognosis. With limited medical resources, it is important to make a difference not only in femoral muscle volume but also in physical function and quality of life. Therefore, the significance of this protocol needs to be further examined in future studies.

## Conclusion

The goals of early mobilization and nutrition therapy were achieved with interventions in the IGREEN protocol, which combined EGDM with NMES and nutrition therapy. This intervention reduced FMV loss in the 10 days after ICU admission.

## Supplementary Information

**Additional file 1: Figure S1.** Daily trajectory of N-titin/Cre. **Table S2.** Results of multivariate linear regression analysis for femoral muscle volume loss. **Figure S3.** Correlation between N-titin/Cre and various muscle strength and physical functions. **Figure S4.** ROC curve for MRC <48 and mean N-titin/Cre.

## Data Availability

The datasets used and/or analysed during the current study are available from the corresponding author on reasonable request.

## Abbreviations

ICU: Intensive care unit
ICU-AW: Intensive care unit-acquired weakness
NMES: Neuromuscular electrical stimulation
EGDM: Early goal-directed mobilization
IGREEN: Intensive goal-directed rehabilitation with electrical muscle stimulation and nutrition
IMS: Intensive care unit mobility scale
EN: Enteral nutrition
MUST: Malnutrition universal screening tool
FMV: Femoral muscle volume
MRC: Medical research council
FSS-ICU: Functional status scores for the intensive care unit
N-titin/Cre: The spot urine N-titin level divided by the spot urine creatinine concentration and by 10
