# Supplementary material for: Novel protocol combining physical and nutrition therapies, Intensive Goal-directed REhabilitation with Electrical muscle stimulation and Nutrition (IGREEN) care bundle
Source: Crit Care. 2021 Dec 4;25:415. doi: 10.1186/s13054-021-03827-8 (PMC8645074; doi:10.1186/s13054-021-03827-8)
Supplement: Supplementary file 1 — Additional file 1: Figure S1. Daily trajectory of N-titin/Cre. Table S2. Results of multivariate linear regression analysis for femoral muscle volume loss. Figure S3. Correlation between N-titin/Cre and various muscle strength and physical functions. Figure S4. ROC curve for MRC <48 and mean N-titin/Cre. [file 13054_2021_3827_MOESM1_ESM.docx]

**Additional File 1**

**The efficacy of the novel concept protocol combining physical and nutrition therapies, Intensive Goal-directed REhabilitation with Electrical muscle stimulation and Nutrition (IGREEN) protocol: A single center historical control study**

Hidehiko Nakano M.D.^1^, Hiromu Naraba M.D.^1^, Hideki Hashimoto M.D. Ph.D.^1^, Masaki Mochizuki M.D.^1^, Yuji Takahashi M.D.^1^, Tomohiro Sonoo M.D.^1^, Yasuhiro Ogawa M.D. Ph.D.^2^, Yujiro Matsuishi R.N. Ph.D.^3^, Nobutake Shimojo M.D. Ph.D.^2^, Yoshiaki Inoue M.D. Ph.D.^2^, Kensuke Nakamura M.D. Ph.D.^1^

1 Department of Emergency and Critical Care Medicine, Hitachi General Hospital, Hitachi, Ibaraki, Japan

2 Department of Emergency and Critical Care Medicine, Faculty of Medicine, University of Tsukuba, Tsukuba, Ibaraki, Japan

3 Neuroscience Nursing, St. Luke’s International University, Tokyo, Japan

***Corresponding Author:**

Hidehiko Nakano

Department of Emergency and Critical Care Medicine, Hitachi General Hospital

2-1-1 Jonancho, Hitachi, Ibaraki, Japan

E-mail: be.rann1988jp@gmail.com

Tel.: +81-294-23-1111, Fax: +81-294-23-8317

**Figure S1 Daily trajectory of N-titin/Cre**


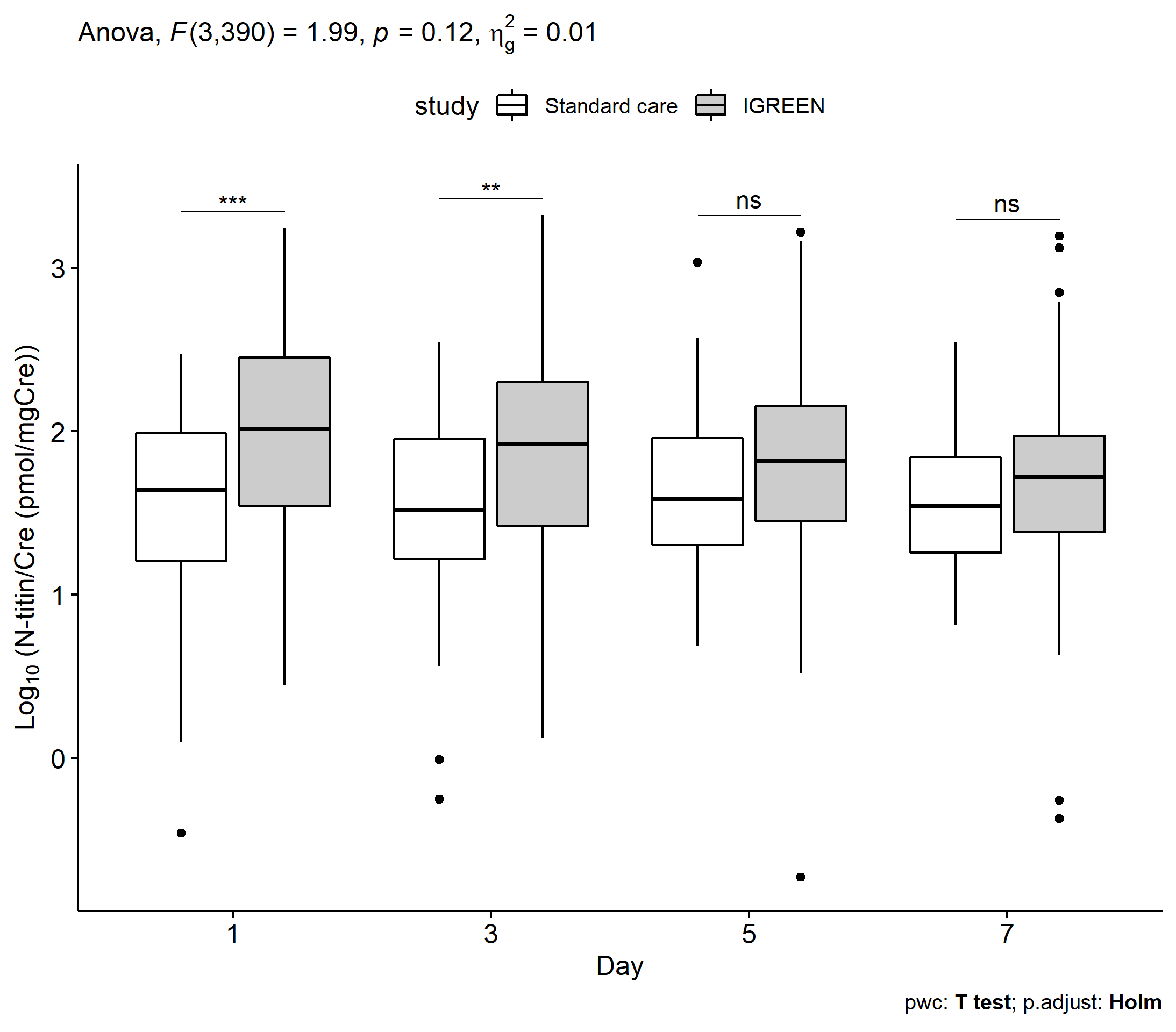


In the box plot, the center line shows the median, the box shows the IQR, and the whisker shows the maximum and minimum. Values less than or greater than 1.5 times the IQR from the first or third quartile are represented by dots as outliers.

***: p < 0.001, **: p< 0.01

N-titin/Cre = titin N-fragment in urine divided by urine creatinine; IGREEN = Intensive goal-directed rehabilitation with electrical muscle stimulation and nutrition protocol; IQR = interquartile range; Anova = analysis of variance; pwc = pair-wise comparison; p.adjust = p value adjustment

**Table S2 Results of multivariate linear regression analysis for femoral muscle volume loss**

| **category** | **Crude Odds** | **p** | **Adjusted Odds** | **p** |
| --- | --- | --- | --- | --- |
| **Age, yr** | 1.00 (0.99–1.01) | 0.78 | 1.06 (0.97–1.15) | 0.2 |
| **Sex, M** | 1.01 (0.81–1.25) | 0.95 | 0.18 (0.01–2.40) | 0.2 |
| **Body height, cm** | 0.99 (0.99–1.00) | 0.26 |  |  |
| **Body weight, kg** | 1.00 (0.99–1.01) | 1.00 |  |  |
| **SOFA** | 1.02 (0.99–1.05) | 0.27 |  |  |
| **APACHE Ⅱ** | 1.01 (1.00–1.03) | 0.12 |  |  |
| **CCI** | 0.95 (0.89–1.00) | 0.07 |  |  |
| **Difficulty walking before admission** | 0.95 (0.67–1.34) | 0.75 |  |  |
| **MUST** | 1.03 (0.93–1.14) | 0.52 |  |  |
| **Diagnosis** | 1.40 (0.82–2.39) | 0.23 |  |  |
| **BUN on day 1, mg/dL** | 1.00 (1.00–1.01) | 0.57 |  |  |
| **Creatinine on day 1, mg/dL** | 0.99 (0.90–1.08) | 0.76 |  |  |
| **Albumin on day 1, mg/dL** | 1.05 (0.89–1.24) | 0.6 |  |  |
| **TLC on day 1, /mm3** | 1.00 (1.00–1.00) | 0.92 |  |  |
| **CRP on day 1, mg/dL** | 1.00 (0.99–1.01) | 0.94 |  |  |
| **mean IMS** | 0.90 (0.86–0.94) | <0.01* | 0.35 (0.18–0.67) | <0.01* |
| **mean Calory delivery, kcal/kg/day** | 0.99 (0.98–1.01) | 0.37 |  |  |
| **mean Protein delivery, g/kg/day** | 1.02 (1.00–1.04) | 0.13 |  |  |
| **Cumulative nitrogen balance** | 1.00 (1.00–1.00) | 0.01* | 0.96 (0.94–0.99) | <0.01* |
| **Duration of MV, d** | 1.04 (1.01–1.07) | 0.01* | 1.19 (0.77–1.85) | 0.43 |
| **RRT use** | 1.08 (0.86–1.35) | 0.53 |  |  |
| **Steroid dose, mg**† | 1.00 (1.00–1.00) | 0.45 |  |  |
| **NMBA use** | 0.60 (0.22–1.62) | 0.32 |  |  |
| **mean N-titin/Cre, pmol/mgCre** | 1.00 (1.00–1.00) | <0.01* | 1.00 (1.00–1.01) | 0.57 |

SOFA = sequential organ failure assessment score; APACHE Ⅱ = acute physiological and chronic health evaluation Ⅱ score; CCI = Charlson Comorbidity Index; MUST = malnutrition universal screening tool; BUN = blood urea nitrogen; TLC = total lymphocyte count; MV = mechanical ventilation; RRT = renal replacement therapy; NMBA = neuromuscular blocking agent; N-titin/Cre = titin N-fragment in urine divided by urine creatinine;

*: p < 0.05, †: Total amount of hydrocortisone equivalent within the 10 days.

**Figure S3 Correlation between N-titin/Cre and various muscle strength and physical functions**


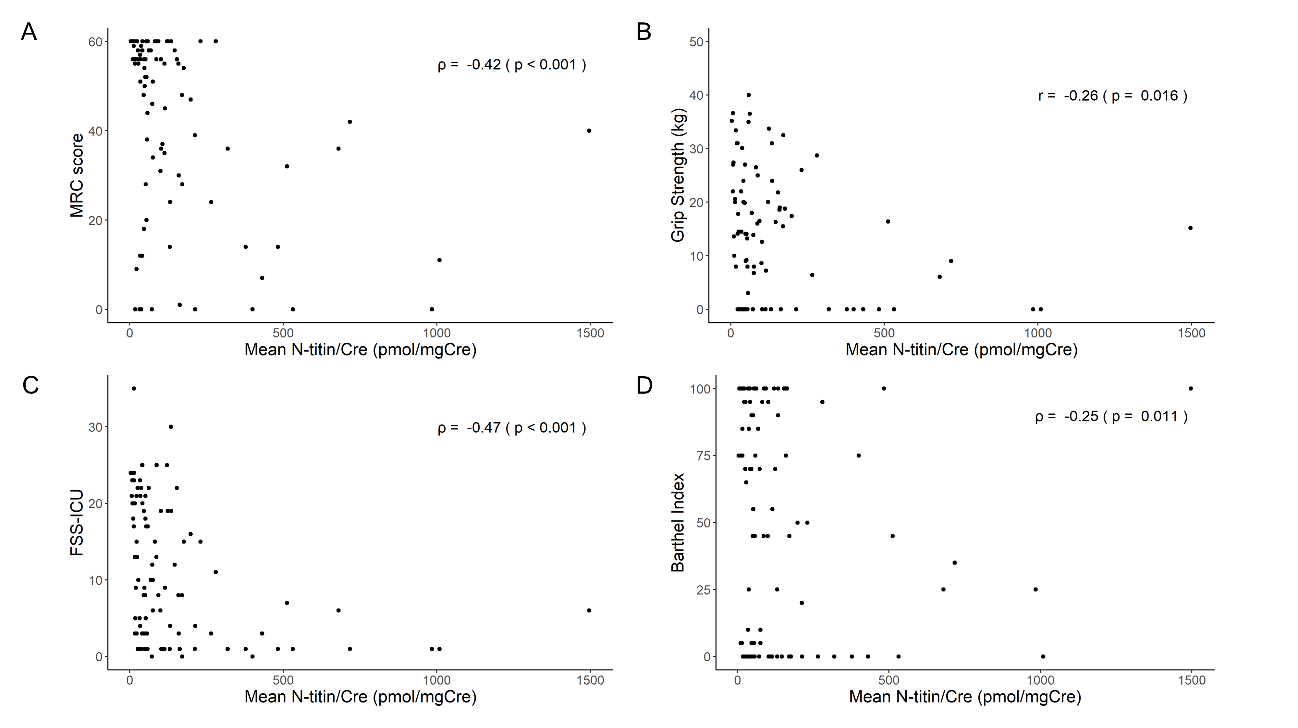


In scatter plots, correlation coefficients calculated by Pearson’s correlation test was shown in r, and those calculated by Spearman's rank correlation test were shown in ρ.

MRC = medical research council; N-titin/Cre = titin N-fragment in urine divided by urine creatinine; FSS-ICU = functional status score for the intensive care unit

**Figure S4 ROC curve for MRC <48 and mean N-titin/Cre**


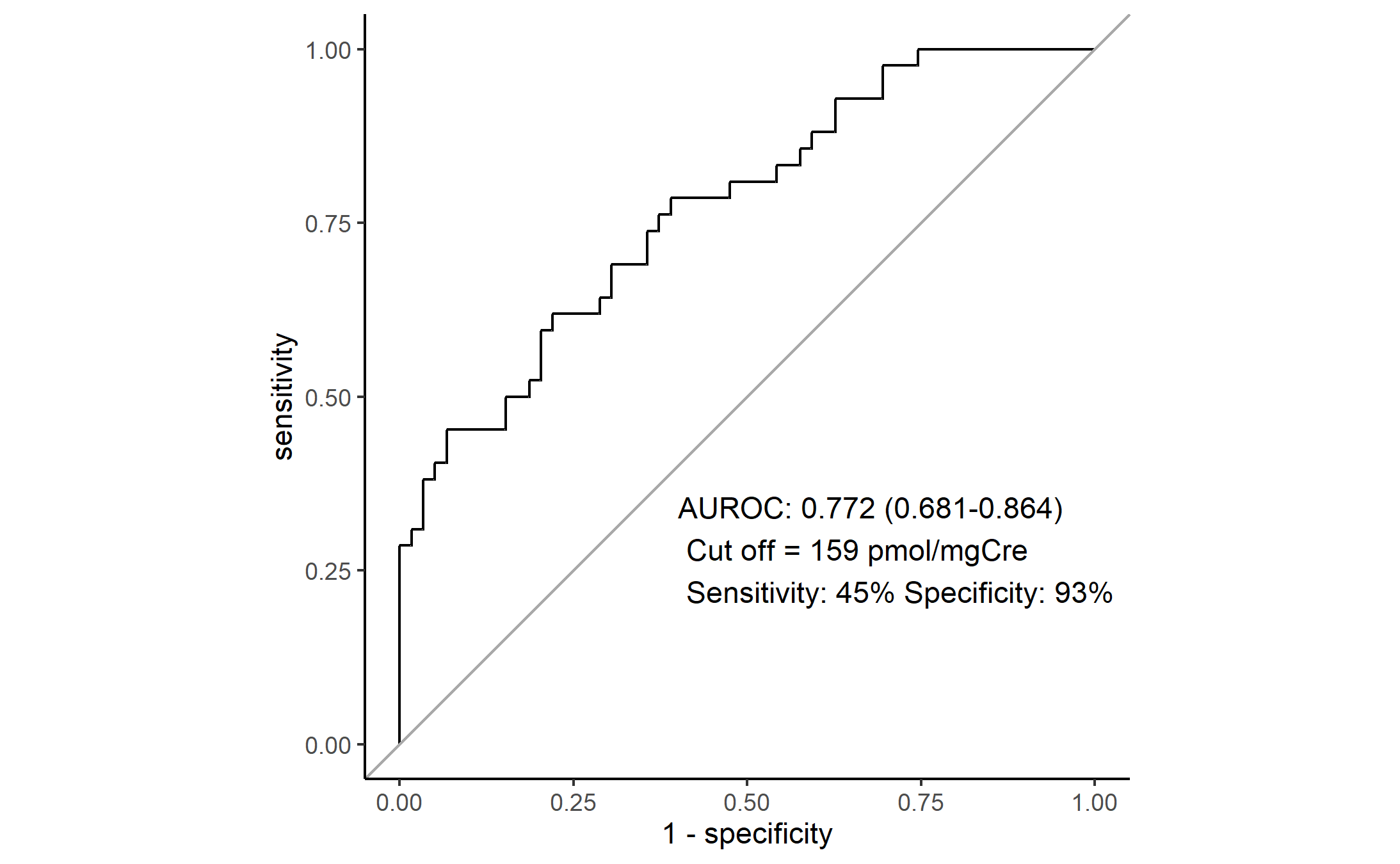


ROC = receiver operating characteristic; MRC = medical research council; N-titin/Cre = titin N-fragment in urine divided by urine creatinine; AUROC = area under receiver operating characteristic
